# Digital image analysis allows objective stratification of patients with silent PIT1‐lineage pituitary neuroendocrine tumors

**DOI:** 10.1002/cjp2.340

**Published:** 2023-09-04

**Authors:** Jiangyan Zhao, Chenxing Ji, Haixia Cheng, Zhen Ye, Boyuan Yao, Ming Shen, Xuefei Shou, Xiang Zhou, Hongying Ye, Zhaoyun Zhang, Hong Chen, Yongfei Wang, Fuchu He, Yao Zhao, Wei Gong, Qilin Zhang, Nidan Qiao

**Affiliations:** ^1^ Department of Neurosurgery, Huashan Hospital, Institutes of Biomedical Sciences Fudan University Shanghai PR China; ^2^ National Center for Neurological Disorders Shanghai PR China; ^3^ Shanghai Key Laboratory of Brain Function and Restoration and Neural Regeneration Shanghai PR China; ^4^ Neurosurgical Institute of Fudan University Shanghai PR China; ^5^ Shanghai Clinical Medical Center of Neurosurgery Shanghai PR China; ^6^ Department of Pathology Huashan Hospital Shanghai PR China; ^7^ Fudan University Graduate School Shanghai PR China; ^8^ Department of Endocrinology Huashan Hospital Shanghai PR China; ^9^ State Key Laboratory of Proteomics, Beijing Proteome Research Center National Center for Protein Sciences Beijing PR China

**Keywords:** artificial intelligence, pituitary adenoma, clustering, nonfunctioning

## Abstract

Studies describing the clinical presentation and prognosis of patients with silent PIT1 (pituitary specific transcription factor)‐lineage pituitary neuroendocrine tumors (PitNETs) are rare. We identified patients with positive PIT1 tumor staining but without evidence of hormone hypersecretion at a tertiary center. Clusters were obtained according to cell morphology and immunostaining from each patient's digitally segmented whole slide image. We compared the clinical presentations, radiological features, and prognoses of the different clusters. We identified 146 patients (68 male, 42.9 ± 14.1 years old) with silent PIT1‐lineage PitNETs. Morphology clustering suggested that tumors with large nuclei and apparent eccentricity were associated with a higher proportion of aggressiveness and a higher hazard of recurrence [hazard ratio (HR): 2.64, (95% CI, 1.06–6.55), *p* = 0.037]. Immunohistochemical clustering suggested that tumors with thyroid stimulating hormone (TSH) staining or all negative PIT1‐lineage hormones were associated with a higher proportion of aggressiveness and a higher risk of recurrence [HR: 12.4, (95% CI, 1.60–93.5), *p* = 0.015]. We obtained three‐tier risk profiles by combining morphological and immunohistochemical clustering. Patients with the high‐risk profile presented the highest recurrence rate compared with those in the medium‐risk and low‐risk profiles [HR: 3.54, (95% CI, 1.40–8.93), *p* = 0.002]. In conclusion, digital image analysis based on cell morphology and immunohistochemical staining allows objective stratification of patients with silent PIT1‐lineage tumors. Typical morphological characteristics of high‐risk tumors are large tumor nuclei and high eccentricity, and typical immunostaining characteristics are TSH staining or negative staining for all PIT1‐lineage hormones.

## Introduction

Clinically nonfunctioning pituitary neuroendocrine tumors (PitNETs) represent a significant portion of pituitary tumors, and the diagnosis is based on excluding hormone hypersecretion. The 2022 World Health Organization (WHO) classification of pituitary tumors advocates the routine use of immunohistochemistry for pituitary transcription factors [PIT1 (pituitary specific transcription factor 1), TPIT (T‐box transcription factor), and SF1 (steroidogenic factor 1)] [1]. Silent corticotropic PitNETs are defined as those with positive TPIT staining but with no evidence of excess cortisol. The 2022 WHO classification and literature reviews also state that PIT1‐lineage PitNETs can be clinically silent [1, 2]. However, studies on the prognosis of silent PIT1‐lineage PitNETs are rare. Therefore, discussing the clinical presentations and prognosis of patients with PIT1 positive tumors but without evidence of hormone hypersecretion is necessary. For patients with functional presentations (hyperprolactinemia, acromegaly, or hyperthyroidism), clinical outcomes mainly focus on medical sensitivity and endocrine remission other than tumor relapse [3, 4].

Among tumors with positive PIT1 staining, the 2022 WHO classification recognizes immature PIT1‐lineage tumors as high‐risk pituitary tumors [1, 5]. Given their aggressive biology, it is important to distinguish these aggressive tumors from other PIT1‐lineage pituitary tumors. However, the definition and diagnostic criteria for immature PIT1‐lineage tumors have varied among studies. Accurate diagnosis based on histology is challenging and may require confirmation with electron microscopy. The ultrastructural hallmark of this subtype is the presence of abundant nuclear spheroids (nuclear inclusions) [6]. However, the presence of spheroids was only observed in half of the patients under electron microscopy in a previous study [7]. Moreover, the presence of spheroids is not specific to any given type of pituitary tumor [8, 9]. Instead, the 2022 WHO classification uses the definition of distinct immunohistochemical staining characterized by focal or scattered positivity for one or more PIT1 family hormones, including growth hormone (GH), prolactin (PRL), and thyroid stimulating hormone (TSH) [1]. The only consistent immunohistochemical finding is diffuse PIT1 positivity. Additionally, a recent study defined plurihormonal PIT1 positivity as positive staining for two of the three PIT1‐lineage hormones (except isolated pairing of GH and PRL) [10].

Confusing diagnostic criteria for immature PIT1‐lineage tumors restrict their clinical application. Here, we attempted to reclassify silent PIT1‐lineage PitNETs using digital image analysis techniques. We compared the clinical presentations, radiological features, and surgical outcomes of patients diagnosed with silent PIT1‐lineage PitNETs and provide an easy‐to‐use classification tool.

## Materials and methods

Consecutive patients with a pathological diagnosis of PitNETs were identified from the Gold Pituitary Database, which recorded all patients with pituitary tumors since 2010 at a tertiary center. All patients underwent surgical tumor resection. The Huashan Hospital Institutional Review Board approved this study, and all patients provided informed consent when their data were logged into the database.

The cohort included patients from 2017, when transcription factor immunostaining was introduced at our center. Immunohistochemistry with primary antibodies was performed using 4‐μm‐thick sections of formalin‐fixed and paraffin‐embedded tissue. Antibodies against the following molecules were used: PIT‐1 (ZSGB‐BIO, Beijing, PR China; ZM‐0208, 1:400), TSH (Long Island Antibody; Shanghai, PR China; M‐0479‐0.2, 1:200), PRL (Long Island Antibody; M‐0451‐0.2, 1:200), GH (Long Island Antibody; M‐0279‐0.2, 1:200), CAM5.2 (Ascend Biotechnology, Luoyang, PR China; AM0380, 1:200), Ki‐67 (Long Island Antibody; M‐0350, 1:200), p53 (Abcam, Boston, MA, USA; Nr. M 7001, 1:50), and SSTR2A (Abcam; ab134152, 1:2,000).

We identified patients with silent PIT1‐lineage PitNETs. The diagnosis was established upon positive PIT1 immunostaining. We excluded patients with symptoms of hormone hypersecretion (including acromegaly and/or hyperthyroidism and/or hyperprolactinemia). We excluded patients with evidence of laboratory hormone hypersecretion (IGF‐1 more than the normal upper limit, elevated free thyroxine with normal or high TSH, or significantly elevated PRL). Patients with mild hyperprolactinemia in the range of the stalk effect were included.

Clinical presentations and radiological features representing the aggressiveness and biological activity of the tumor were identified, including multiple surgical histories (more than twice), giant tumor (defined as maximal tumor diameter larger than 4 cm), sphenoidal/clivus extension, Knosp grade, suprasellar extension (lateral, anterior, or superior), high Ki‐67 (defined as a Ki‐67 index higher than 5%), tenacious texture (necessitating scissors to dissect the tumor during surgery), and subtotal resection (obvious tumor residue on postoperative imaging). Pathologists' reports were also documented.

Hematoxylin and eosin (H&E) and immunohistochemically stained slices (GH, PRL, and TSH) were converted into whole slide images (WSIs) using a Panoramic 250 FLASH/MIDI Scanner (3DHISTECH, Budapest, Hungary) at a ×40 resolution. The WSIs were processed using QuPath software 0.2.2 [11] and analyzed using the same method as in a previous study [12]. In brief: (1) a built‐in stain vector estimator preprocessed the images. A trained pixel classifier was built with random trees to distinguish the ‘tumor’, ‘stroma’, ‘hemorrhage’, and ‘background area’. These areas were first annotated by an experienced pathologist in 15 cases. (2) Tumor cells were identified by built‐in cell detection using nuclear staining (hematoxylin). The mean diaminobenzidine (DAB) optical density (OD) of the nucleus was subtracted from the mean DAB OD of the cytoplasm to reduce false positive cells. (3) The WSI analysis protocol scripts were created and batch processed for each set of images. The automatically segmented areas, ‘tumor’, ‘stroma’, and ‘hemorrhage’ were then validated in 20 randomly selected slides annotated by an experienced pathologist.

### Morphology classification

**Figure 1:**
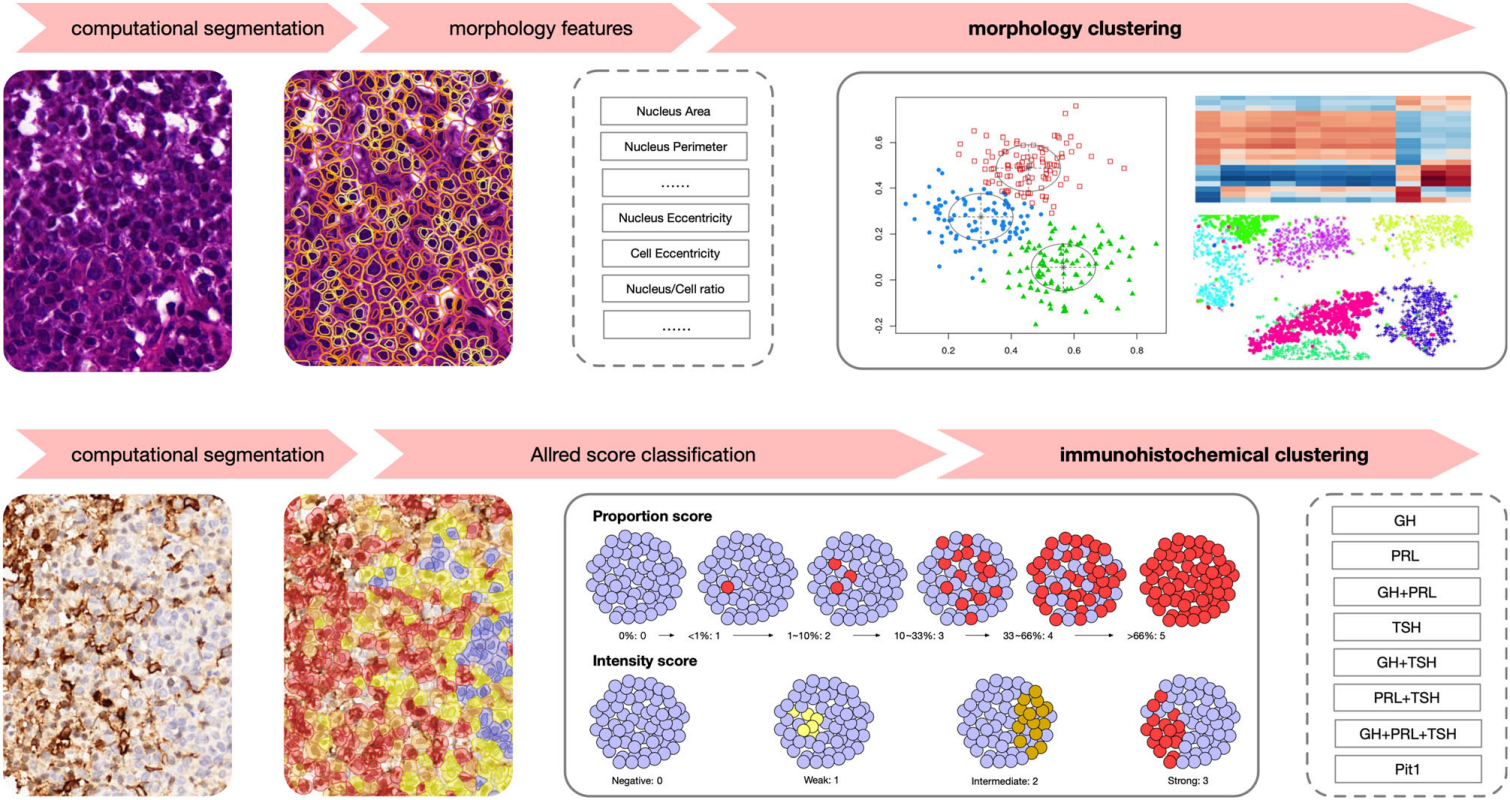
Study workflow. Digital segmentation was used to segment cells based on H&E‐stained WSIs (with the ground truth first annotated by pathologists). Staining intensity was calculated based on immunohistochemical WSIs (purple, negative; yellow, weak; brown, intermediate; and red, strong).

Cell nuclei within the tumor area were automatically segmented (Figure 1), where six nucleus features, including ‘Nucleus Area’, ‘Nucleus Perimeter’, ‘Nucleus Circularity’, ‘Nucleus Maxcaliper’, ‘Nucleus Mincaliper’, and ‘Nucleus Eccentricity’, were calculated. Similarly, six cell features, including ‘Cell Area’, ‘Cell Perimeter’, ‘Cell Circularity’, ‘Cell Maxcaliper’, ‘Cell Mincaliper’, and ‘Cell Eccentricity’, were calculated. ‘Nucleus/Cell ratio’ was also extracted. The extracted features were then aggregated by the 25th and 75th quantile values. In total, 39 features were generated for each patient. We used an unsupervised clustering method based on Gaussian finite mixture models [13] to generate clusters with different morphologies. A model with the lowest Bayesian information criteria was selected over other models. We visually checked cluster similarity using correlation heatmaps and t‐distributed stochastic neighbor embedding (t‐SNE) plots.

### Immunohistochemical classification

Because both staining area and intensity are important in classifying PIT1‐lineage tumors, we adopted the Allred score [14], which combines both the proportion score (percentage of positive cells) and the intensity score (intensity of the reaction) to determine the positive or negative staining of GH, PRL, or TSH (Figure 1). The scores were then added to obtain a final score with eight possible values. Scores of more than 3 were considered positive. Considering the entrapped pituitary cells may have affected the immunostaining quantifications, an additional criterion of >5% stained cells was used as a cutoff for positivity. According to the results, these tumors were further categorized into eight immunohistochemical subtypes [GH (only GH positive), PRL (only PRL positive), TSH (only TSH positive), GH + TSH (both GH and TSH positive), GH + PRL (both GH and PRL positive), PRL + TSH (both PRL and TSH positive), GH + PRL + TSH (all three positive), and Pit1 (all three negative)]. Immunostaining clusters (ICs) were generated by observing the distribution of clinical aggressiveness.

The percentage‐based method calculated the percentage of positive tumor cells in each immunohistochemical staining by normalizing the cell number of tumor areas in each slide. More than 10% were considered positive.

The *H*‐score‐based method was calculated by multiplying the percentage of cells demonstrating each intensity (‘negative’, ‘weak’, ‘intermediate’, and ‘strong’, scored from 0 to 3) and adding the results. *H*‐score more than 5 was considered positive.

### WHO classification

We determined the subclassification of Pit1‐lineage tumors according to GH, PRL, and TSH immunostaining. The pathologists referred the 2022 WHO classification [1] and discussed the subclassifications.

### Statistical analysis

Continuous data with normal distributions are displayed as mean ± standard deviation; otherwise, median values with interquartile ranges are displayed. We compared the clinical presentations, radiological features, and recurrence probabilities between the different morphologies and immunostaining clusters. The final risk profiling was performed by combining morphological and immunostaining clusters. We used chi‐square tests for categorical variables, *t*‐tests for continuous variables, and Kaplan–Meier analysis for relapse probability among different groups. All statistical analyses were performed using R software, version 3.4.2 (https://www.r-project.org).

## Results

Among the 12,045 patients with PitNETs in the database, we identified 146 (68 male, 42.9 ± 14.1 years old) with silent PIT1‐lineage PitNETs (Table 1). Ten patients underwent repeated operations and suffered recurrent disease. Sixteen patients had Knosp grade IV tumors, and 19 had invasive tumors laterally, anteriorly, or superiorly. Nineteen patients were classified as having giant pituitary tumors with a maximum diameter of more than 4 cm. Seven patients had a Ki‐67 index of more than 5%.

**Table 1:** Overall characteristics of the cohort

	Overall (*N* = 146)
Age (years)	42.9 (14.1)
Gender (female)	78 (53.4%)
Symptoms	
Incidental	38 (26.0%)
Visual defect	62 (42.5%)
Headache	47 (32.2%)
Radiotherapy history	6 (4.1%)
Surgical history	
None	125 (85.6%)
Once	11 (7.5%)
More than once	10 (6.9%)
Knosp grade	
I–II	100 (68.5%)
III	30 (20.6%)
IV	16 (10.9%)
Extrasellar invasion	
Sphenoidal sinus	11 (7.5%)
Clivus	8 (5.5%)
Lateral	6 (4.1%)
Superior	9 (6.2%)
Anterior	5 (3.4%)
Giant tumor (≥4 cm)	19 (13.0%)
WHO subclassification	
Somatotroph	29 (19.9%)
Lactotroph	10 (6.9%)
Mammosomatotroph	19 (13.0%)
Thyrotroph	9 (6.2%)
Mature plurihormonal PIT1 lineage	38 (26.0%)
Immature PIT1 lineage	26 (17.8%)
PitNET, NOS	15 (10.3%)

### Morphology classification

**Figure 2:**
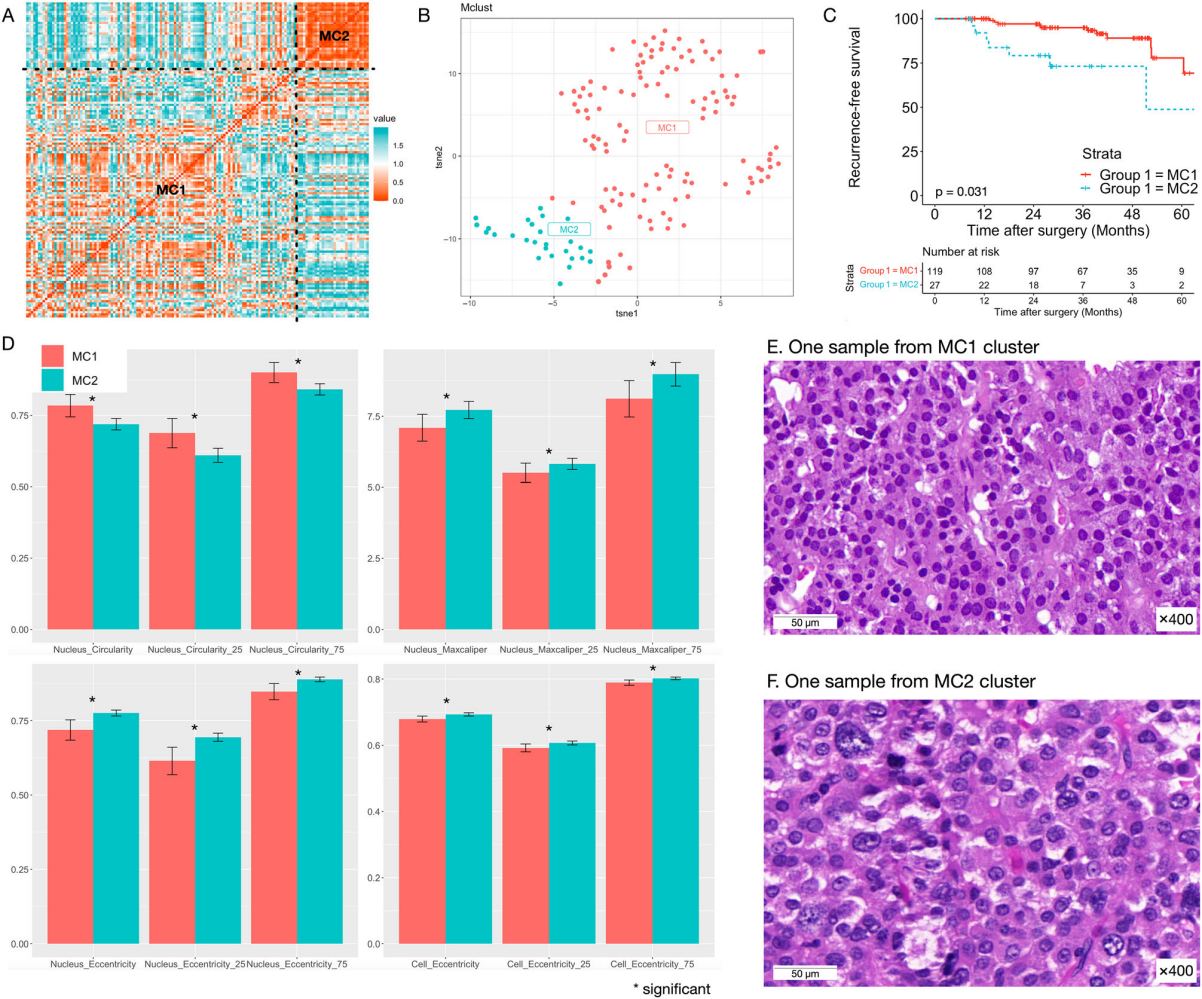
Morphology clustering. (A) Distance heatmap showing a cluster of samples in the right upper part (value means correlation distance, lies from 0 to 2; measures the linear relationship between the two vectors; red indicates close and blue indicates far). (B) t‐SNE plot showing two major clusters (MC1 and MC2). (C) Kaplan–Meier analysis showing high relapse probability in MC2. (D) Morphology features of significant differences in the two MCs. (E) One sample from MC1 shows regular‐shaped tumor cells. (F). One sample from MC2 shows a large tumor nucleus and obvious nuclear pleomorphism.

An unsupervised machine learning algorithm identified two major morphology clusters (MCs, 119 and 27 in each) based on the H&E‐stained WSI. Samples were near (with a small distance, 0.220) to each other if they were in the same cluster and far (with a large distance, 1.182) from each other if they were in different clusters (Figure 2A). The spatial distribution is shown in the t‐SNE plot (Figure 2B).

The proportions of giant tumors, sphenoidal/clivus extension, Knosp grades III and IV, and suprasellar extension were higher (33.3% versus 8.4%, 25.9% versus 5.9%, 55.6% versus 26.0%, and 37.4% versus 7.6%, respectively) in MC2 tumors than in MC1 tumors (Table 2). Tumors in MC2 were also associated with a higher hazard of recurrence [hazard ratio (HR): 2.64, (95% CI, 1.06–6.55), *p* = 0.037]. The 4‐year recurrence‐free survival was 89.0% (95% CI, 81.6–97.1%) in MC1 tumors and 48.8% (95% CI, 21.0–94.7%) in MC2 tumors (Figure 2C).

**Table 2:** Characteristics of tumors using different classifications

	Morphology classification			Immunohistochemical classification		
	MC1 (*N* = 119)	MC2 (*N* = 27)	*p*	IC1 (*N* = 68)	IC2 (*N* = 78)	*p*
Age (years)	43.6 (14.2)	39.3 (13.2)	0.149	43.5 (13.2)	42.3 (14.9)	0.636
Gender (female)	68 (57.1%)	10 (37.0%)	0.094	33 (48.5%)	45 (57.7%)	0.347
Multiple surgeries	5 (4.2%)	5 (18.5%)	0.069	1 (1.5%)	9 (11.6%)	0.001
Symptoms						
Incidental	31 (26.1%)	7 (25.9%)	1.000	21 (30.9%)	17 (21.8%)	0.289
Visual defect	47 (39.5%)	15 (55.6%)	0.191	25 (36.8%)	37 (47.4%)	0.257
Headache	41 (34.5%)	6 (22.2%)	0.317	19 (27.9%)	28 (35.9%)	0.396
Radiological findings						
Giant tumor	10 (8.4%)	9 (33.3%)	0.002	1 (1.5%)	18 (23.1%)	<0.001
Sphenoidal/clivus extension	7 (5.9%)	7 (25.9%)	0.005	5 (7.4%)	9 (11.5%)	0.565
Knosp grade III–IV	31 (26.0%)	15 (55.6%)	0.001	18 (26.4%)	28 (35.7%)	0.267
Suprasellar extension	9 (7.6%)	10 (37.4%)	<0.001	5 (7.4%)	14 (18.0%)	0.099
Pathological findings						
High Ki‐67 index (>5%)	4 (3.4%)	3 (11.1%)	0.229	1 (1.5%)	6 (7.7%)	0.172
Positive p53 staining	53 (44.5%)	10 (37.0%)	0.696	31 (56.4%)	32 (51.6%)	0.742
Positive SSTR2a staining	84 (70.6%)	20 (74.2%)	0.320	50 (92.6%)	54 (91.5%)	1.000
Surgical findings						
Tenacious consistency	32 (26.7%)	9 (33.3%)	0.663	12 (17.7%)	29 (37.2%)	0.015
Subtotal resection	19 (16.0%)	7 (25.9%)	0.346	9 (13.2%)	17 (21.8%)	0.258
WHO subclassification			<0.001			<0.001
Somatotroph	29 (24.4%)	0 (0.0%)		24 (35.3%)	5 (6.4%)	
Lactotroph	7 (5.9%)	3 (11.1%)		8 (11.8%)	2 (2.7%)	
Mammosomatotroph	17 (14.3%)	2 (7.4%)		15 (22.1%)	4 (5.1%)	
Thyrotroph	9 (7.6%)	0 (0.0%)		2 (3.0%)	7 (9.5%)	
Mature plurihormonal PIT1 lineage	38 (31.9%)	0 (0.0%)		11 (16.2%)	27 (36.5%)	
Immature PIT1 lineage	14 (11.8%)	12 (44.4%)		6 (8.8%)	9 (12.2%)	
PitNET, NOS	5 (4.2%)	10 (37.0%)		2 (3.0%)	24 (32.4%)	

We then compared the features of MC1 and MC2 tumors. After adjusting for multiple comparisons (supplementary material, Table S1), features that were statistically significant in the mean, 25th quantile, and 75th quantile included nucleus circularity, nucleus maxcaliper, nucleus eccentricity, and cell eccentricity (Figure 2D). MC2 tumors had lower nucleus circularity, higher nucleus maxcaliper, nucleus eccentricity, and cell eccentricity, suggesting that larger tumor nuclei and obvious nuclear pleomorphism were key features of MC2 tumors (Figure 2E,F).

### Immunohistochemical classification

**Figure 3:**
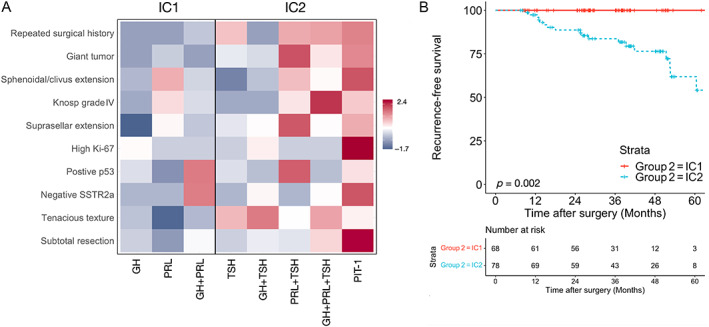
Immunohistochemical clustering. (A) Matrix showing the proportion of tumor aggressiveness and biological activity in different immunohistochemical subtypes (blue represents low, indicating relative low proportion in the same row; red represents high, indicating relative high proportion in the same row; the number was calculated by subtracting the mean and dividing by the standard deviation). (B) Kaplan–Meier analysis showing a high relapse probability in IC2.

Immunostaining and Allred score‐based classification assigned 24, 13, 31, 8, 20, 7, 20, and 23 cases as GH, PRL, GH + PRL, TSH, GH + TSH, PRL + TSH, GH + RPL + TSH, and Pit‐1 subtypes, respectively (supplementary material, Table S2). Figure 3A and Table 2 show that the GH, PRL, and GH + PRL subtypes (IC1) present a lower proportion of aggressiveness and biological activity than the other subtypes (IC2). Tumors in IC2 were also associated with a higher hazard of recurrence (HR: 12.4, (95% CI, 1.60–93.5), *p* = 0.015; Figure 3B).

### Combining both morphology and immunostaining classification

**Figure 4:**
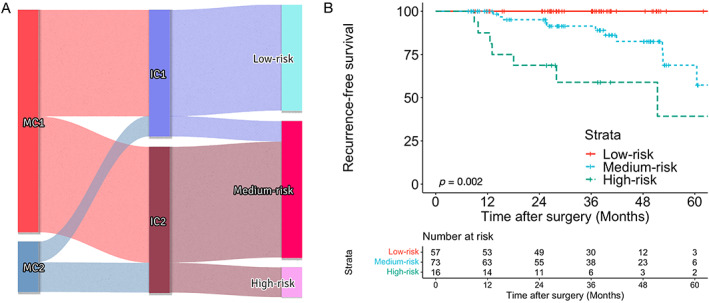
Combined risk profile. (A) Sankey plot showing the three‐tier risk profile generated by combining morphology and immunohistochemical clustering. (B) Kaplan–Meier analysis showing the highest relapse probability in the high‐risk profile.

We obtained three risk profiles by combining morphology and immunostaining classification (Figure 4A). The low‐risk profile was defined as both morphological and immunostaining clusters in the low‐risk range (MC1 and IC1). A medium‐risk profile was defined as either morphological or immunostaining clusters in the low‐risk range (MC1, IC2, MC2, and IC1). A high‐risk profile was defined as both morphological and immunostaining clusters in the high‐risk range (MC2 and IC2). Patients in the high‐risk profile presented with more tumor aggressiveness (Table 3) and were associated with the highest recurrence rate compared with those in the medium‐risk and low‐risk groups (HR: 3.54, (95% CI, 1.40–8.93), *p* = 0.002).

**Table 3:** Characteristics of tumors in the combined clusters

	Low‐risk (*N* = 57)	Medium‐risk (*N* = 73)	High‐risk (*N* = 16)	*p*
Age (years)	43.1 (13.8)	44.4 (14.0)	35.2 (13.9)	0.059
Gender (female)	30 (52.6%)	41 (56.2%)	7 (43.8%)	0.658
Multiple surgeries	1 (1.8%)	4 (5.5%)	5 (31.3%)	<0.001
Symptoms				
Incidental	15 (26.3%)	22 (30.1%)	1 (6.3%)	0.143
Visual defect	21 (36.8%)	30 (41.1%)	11 (68.8%)	0.070
Headache	18 (31.6%)	24 (32.9%)	5 (31.3%)	0.984
Radiological findings				
Giant tumor	1 (1.8%)	9 (12.3%)	9 (56.3%)	<0.001
Sphenoidal/clivus extension	3 (5.3%)	6 (8.2%)	5 (31.3%)	0.007
Knosp grade III–IV	13 (22.8%)	23 (31.5%)	10 (62.5%)	0.051
Suprasellar extension	2 (3.5%)	10 (13.7%)	7 (43.8%)	<0.001
Pathological findings				
High Ki‐67 index (>5%)	1 (1.8%)	3 (4.1%)	3 (18.8%)	0.018
Positive p53 staining	28 (49.1%)	28 (38.4%)	7 (43.8%)	0.718
Positive SSTR2a staining	44 (77.2%)	46 (63.0%)	14 (87.5%)	0.483
Surgical findings				
Tenacious consistency	11 (19.3%)	22 (30.1%)	8 (50.0%)	0.047
Subtotal resection	8 (14.0%)	12 (16.4%)	6 (37.5%)	0.087

### Comparison between the digital and pathologists' diagnoses

We compared immunohistochemical classifications made by the pathologists and the classifications determined by the Allred score. We found that the correlation was only 48.6% (95% CI 40.3–57.0%) between the two diagnoses and the confusion matrix is presented in supplementary material, Figure S1. We tested the inter‐observer reliability of diagnosing an immature PIT1‐lineage tumor in our center by two pathologists and found that the inter‐observer coefficient was only 0.703, suggesting that different pathologists did not agree on the diagnosis. A final diagnosis of the specific subclassification was determined after discussion. Immature PIT1‐lineage tumors presented with more tumor aggressiveness and were associated with the highest recurrence rate (supplementary material, Table S3). Among 16 cases classified in the high‐risk profile, only 12 were diagnosed as ‘immature PIT1‐lineage tumor’ and the other 4 were concluded as an ‘undetermined’ [not otherwise specified (NOS)] diagnosis. Among the other 130 cases with a low‐risk or medium‐risk profile, 14 were diagnosed as ‘immature PIT1‐lineage tumor’ and 11 were ‘undetermined’ (NOS) (supplementary material, Figure S2).

### Comparison with other PitNETs

The overall prognosis of patients in the whole population was worse than that of those with SF1‐positive tumors (108 patients in the same database), with a 4‐year recurrence‐free survival rate of 85.8% (95% CI, 78.6–93.8%) and 97.2% (95% CI, 94.2–100%). However, the prognosis of tumors in the low‐risk and medium‐risk profile [4‐year recurrence‐free survival rate 95.0% (95% CI, 90.7–99.4%)] was similar to that of those with SF1‐positive tumors.

## Discussion

**Figure 5:**
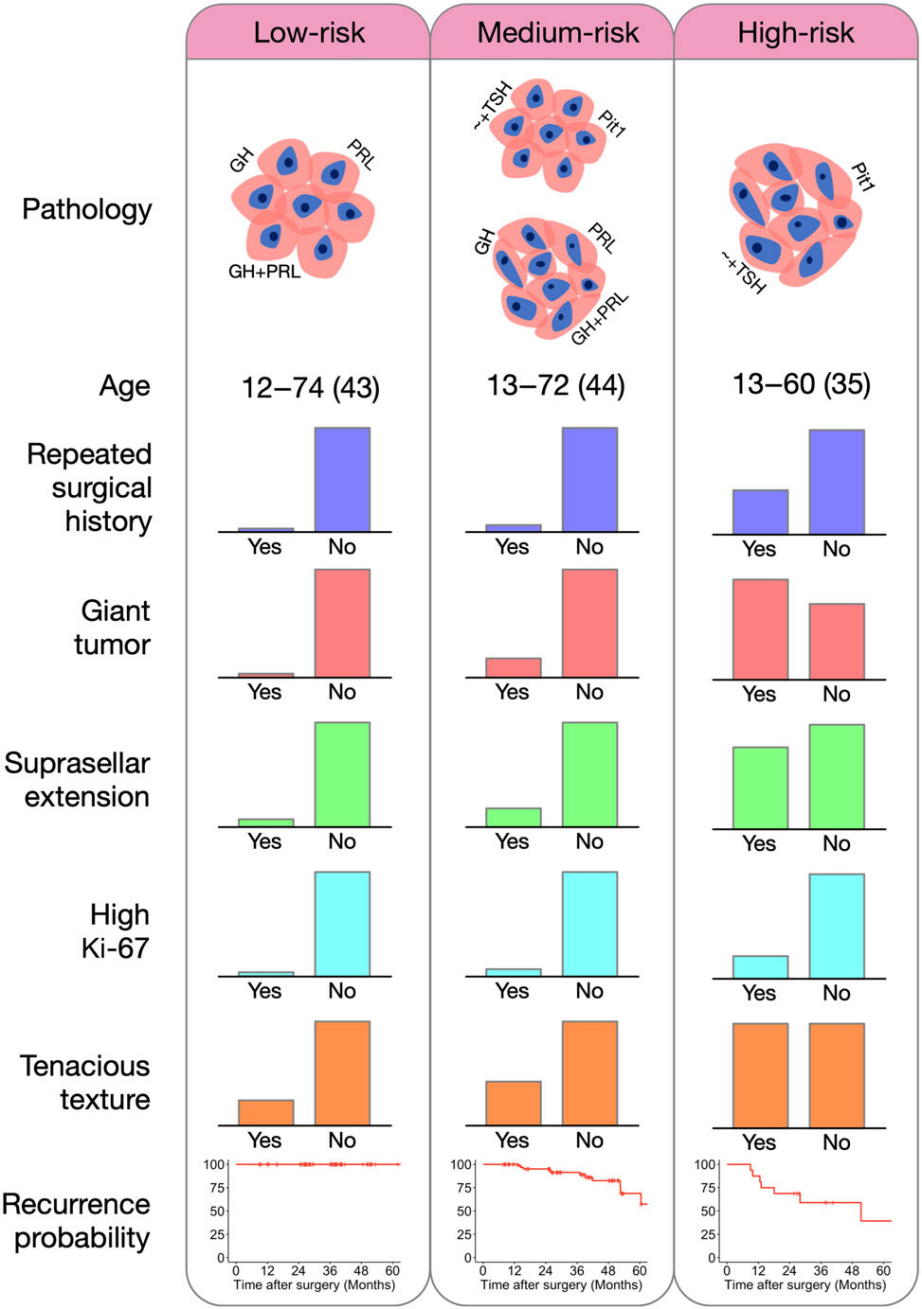
New classification scheme to identify high‐risk tumors in patients with silent PIT1‐lineage PitNETs.

In this study, we describe the clinical presentations, radiological features, and prognosis of subtypes of silent PIT1‐lineage PitNETs. Accordingly, we propose a new classification scheme to identify high‐risk tumors in patients with silent PIT1‐lineage PitNETs (Figure 5), which is in line with the current WHO classification.

According to the 2022 WHO classification, clinically nonfunctional pituitary adenomas include SF1‐lineage tumors, silent TPIT‐lineage tumors, null cell tumors, and some PIT1‐lineage tumors. Immature PIT1‐lineage tumors are common in younger patients [1]. However, not all patients with a PIT1‐lineage adenoma fall into the category of ‘immature’. Other types of silent PIT1‐lineage adenoma present with benign behavior, as the low‐risk group demonstrated in our study.

The diagnostic criteria and nomenclature of immature PIT1‐lineage tumors are changing and inconsistent among different studies, impeding their clinical usage [6, 7, 8, 9, 10, 15, 16]. The 2022 WHO guidelines consider immature PIT1‐lineage tumors with the following features: nuclear pleomorphism, prominent nuclear inclusions, diffuse nuclear PIT1 positivity, and variable GH, PRL, and/or TSH in any combination. However, these criteria are subjective and depend on the pathologist's experience. This could explain the low inter‐observer reliability and the inconsistency between the pathologists' diagnosis and the digital diagnosis.

Digital morphology clustering identifies that tumors with a high probability of recurrence are associated with large tumor nuclei and high eccentricity. This result is similar to that of previous studies, which suggested that immature PIT1‐lineage tumors are composed of spindle and polygonal or even spindle‐shaped cells with nuclear pleomorphism [8, 9]. In our study, we used the Allred score to determine the status of immunohistochemical staining and to provide a more objective assessment. The Allred score is one of the most promising semiquantitative composite scoring systems. Previous studies have suggested that the Allred score helps predict the efficacy of hormonal therapy in patients with breast and endometrial cancer [17, 18]. Pathologists' classifications of the immunostaining were retrieved from reports issued by different pathologists. Discussion by a board of experienced pathologists could have increased the unexpected low correlation between the pathologists' diagnosis and the digital diagnosis. We also compared the HRs in differentiating recurrence risk using *H*‐score‐based and proportion‐based classifications and found that these two methods were inferior to the Allred score (supplementary material, Table S4).

The high‐risk profile in our study is the nearest match to ‘immature PIT1‐lineage tumors’ in the WHO 2022 classification. Previous studies have reported that these tumors are clustered much younger than other common clinical nonfunctioning tumors. Similarly, we observed younger ages in the high‐risk profile. The subtotal resection rate and recurrence are similar to previous reports stating that these tumors had high invasion and high recurrence probability (37–53% had progression) [6, 7, 8, 9, 10, 16]. Overall, the clinical presentations, radiological features, and prognosis of our high‐risk profile are comparable with the reported ‘immature’ type, suggesting that our method could serve as an alternative and objective assessment tool.

We further investigated the microenvironment of different risk profiles. We used a cell detection algorithm to segment the tumor, stroma, and blood within the H&E‐stained WSI and calculated the proportion of each component. We found that tumors in the medium‐ and high‐risk groups were associated with a higher proportion of stroma (supplementary material, Figure S3), suggesting that the tumor microenvironment might significantly mediate aggressiveness and biological activity in patients with silent PIT1‐lineage tumors [19, 20].

Our approach could be written into an automated script and be potentially integrated into a clinical decision support system for pituitary tumor diagnosis. However, this study has several limitations. First, the clinical term ‘nonfunctioning’ indicates only a lack of hormone hypersecretion. Patients may suffer from hypopituitarism and are not considered ‘clinically silent’ in some expert endocrinological practices. Second, this is a single center study, and the proposed clustering method should be further validated in an external center with different sampling methods. For example, the amount of entrapped pituitary cells in the WSI might disturb the accuracy of the digital immunostaining quantifications. Though we set 5% as the cutoff for positivity, staining of pituitary cells could not be excluded in certain slides. Third, the comparison between silent PIT1‐lineage tumors and SF1‐lineage tumors is preliminary. Finally, we did not discuss the clinical presentations and prognosis of patients with functional PIT1‐lineage tumors because the treatment modality and outcome assessment differ from those of patients with clinically silent tumors.

In conclusion, silent PIT1‐lineage PitNETs are defined as clinically nonfunctional tumors with positive PIT1 staining. Digital image analysis classification provides stratification to differentiate between aggressiveness and recurrence risk in these patients. Typical morphological characteristics of high‐risk tumors are large tumor nuclei and high eccentricity, and typical immunostaining characteristics are TSH staining or negative staining for all PIT1‐lineage hormones.

## Author contributions statement

CJ, JZ and HC performed the analyses and wrote the first draft of the manuscript. NQ, YZ and QZ designed this study. CJ, HC, QZ, and BY collected the data. HY and ZZ provided endocrinological consultations for this study. MS, XS and YW provided neurosurgical consultations for this study. HC, FH, YZ and NQ revised the draft, and the final version was approved by all listed authors.

## Ethics approval and consent to participate

The institutional review board of Huashan Hospital approved the study. Patients provided informed consent when their data were logged into the database.

## Supporting information


**Figure S1.** Confusion matrix of the Allred score‐based immunostaining classification versus pathologists' classification. Value means case count in the corresponding *x*‐axis and the *y*‐axis
**Figure S2.** Confusion matrix of the digital classification versus 2022 WHO classification. Value means case count in the corresponding *x*‐axis and the *y*‐axis
**Figure S3.** Tumors in the medium‐risk and high‐risk groups were associated with a higher proportion of stroma
**Table S1.** Feature comparison in morphology clusters (MC)1 and 2
**Table S2.** Characteristics among different immunostaining subtypes
**Table S3.** Characteristics among different WHO subclassification
**Table S4.** Hazard ratio using other classification methods based on immunostaining

QuPath script

## Data Availability

The QuPath script is provided as a supplementary file. All analyses were performed using R, and the Python code is available upon request.
