# Supplementary material for: Digital image analysis allows objective stratification of patients with silent PIT1‐lineage pituitary neuroendocrine tumors
Source: J Pathol Clin Res. 2023 Sep 4;9(6):488–97. doi: 10.1002/cjp2.340 (PMC10556262; doi:10.1002/cjp2.340)
Supplement: Supplementary file 1 — Figure S1. Confusion matrix of the Allred score‐based immunostaining classification versus pathologists' classification. Value means case count in the corresponding x‐axis and the y‐axis Figure S2. Confusion matrix of the digital classification versus 2022 WHO classification. Value means case count in the corresponding x‐axis and the y‐axis Figure S3. Tumors in the medium‐risk and high‐risk groups were associated with a higher proportion of stroma Table S1. Feature comparison in morphology clusters (MC)1 and 2 Table S2. Characteristics among different immunostaining subtypes Table S3. Characteristics among different WHO subclassification Table S4. Hazard ratio using other classification methods based on immunostaining [file CJP2-9-488-s001.pdf]

Digital image analysis allows objective stratification of patients with silent PIT1-lineage pituitary neuroendocrine tumors

J Zhao, C Ji, H Cheng, Z Ye *et al*., *J Pathol Clin Res,* https://doi.org/10.1002/cjp2.340

Supplementary Figures S1–S3

Supplementary Tables S1–S4

QuPath script. Provided as a separate file

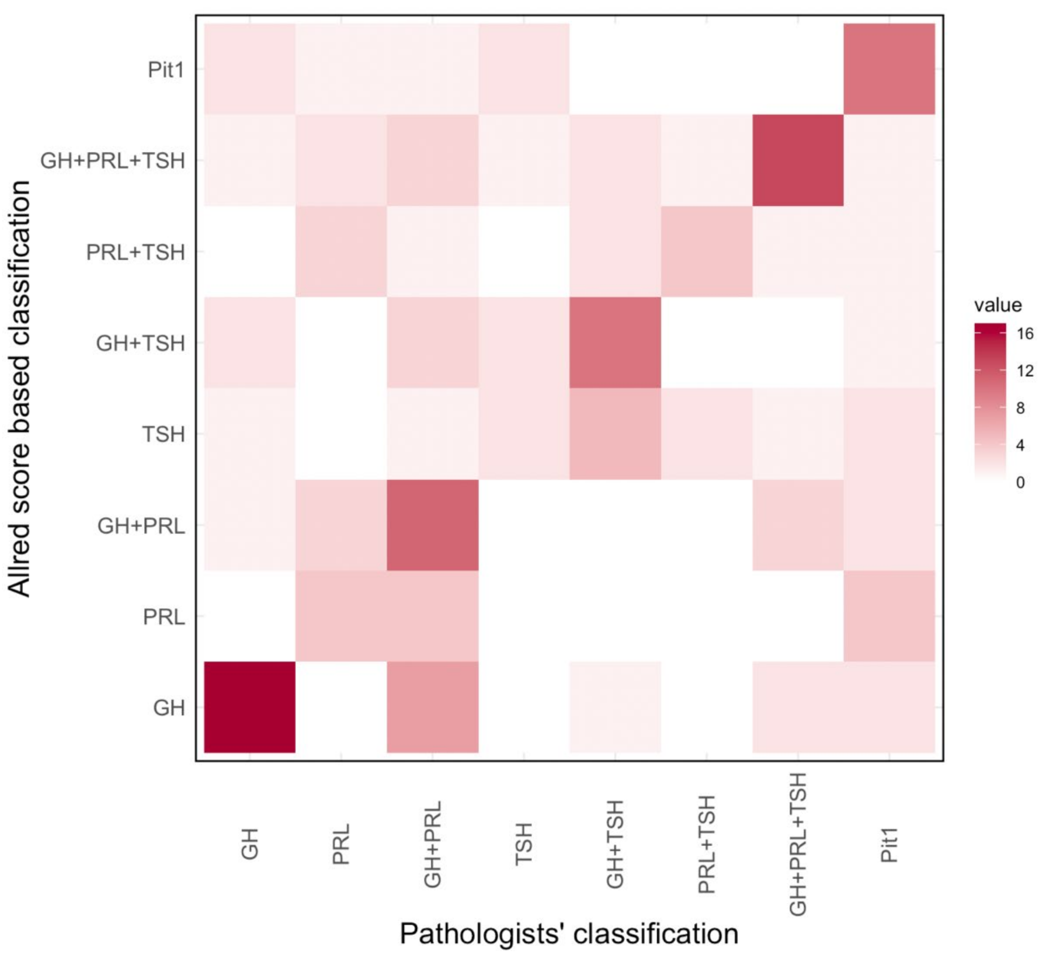

Figure S1. Confusion matrix of the Allred score-based immunostaining classification versus pathologists' classification. Value = case count in the corresponding *x*-axis and *y*-axis.

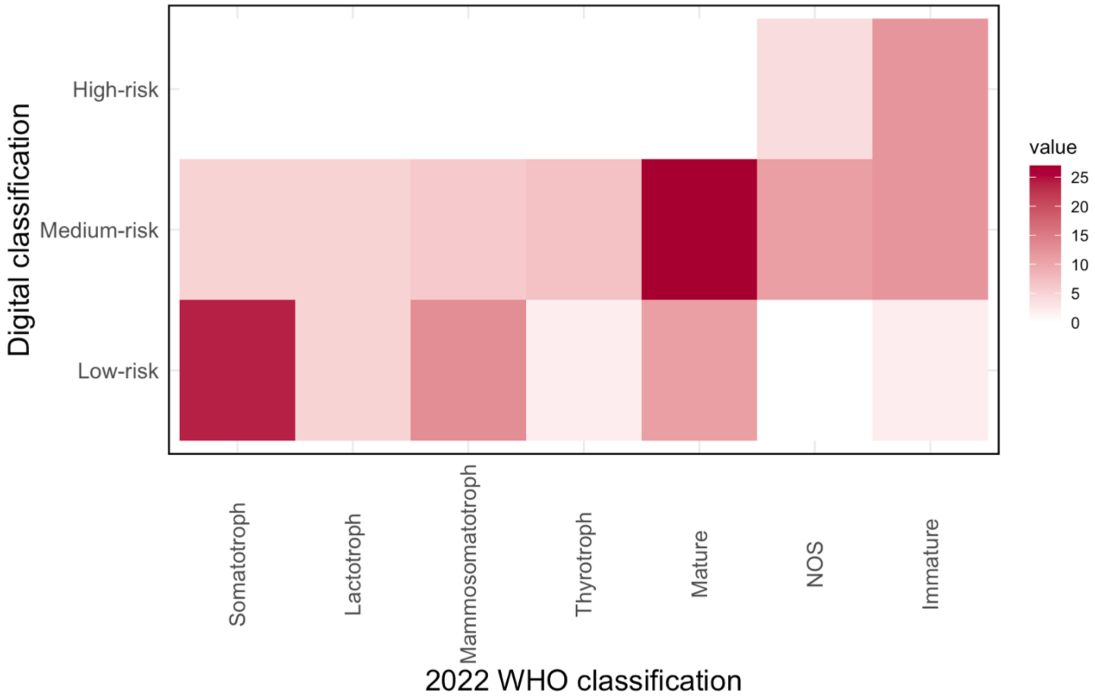

Figure S2. Confusion matrix of the digital classification versus the 2022 WHO classification. Value = case count in the corresponding *x*-axis and *y*-axis.

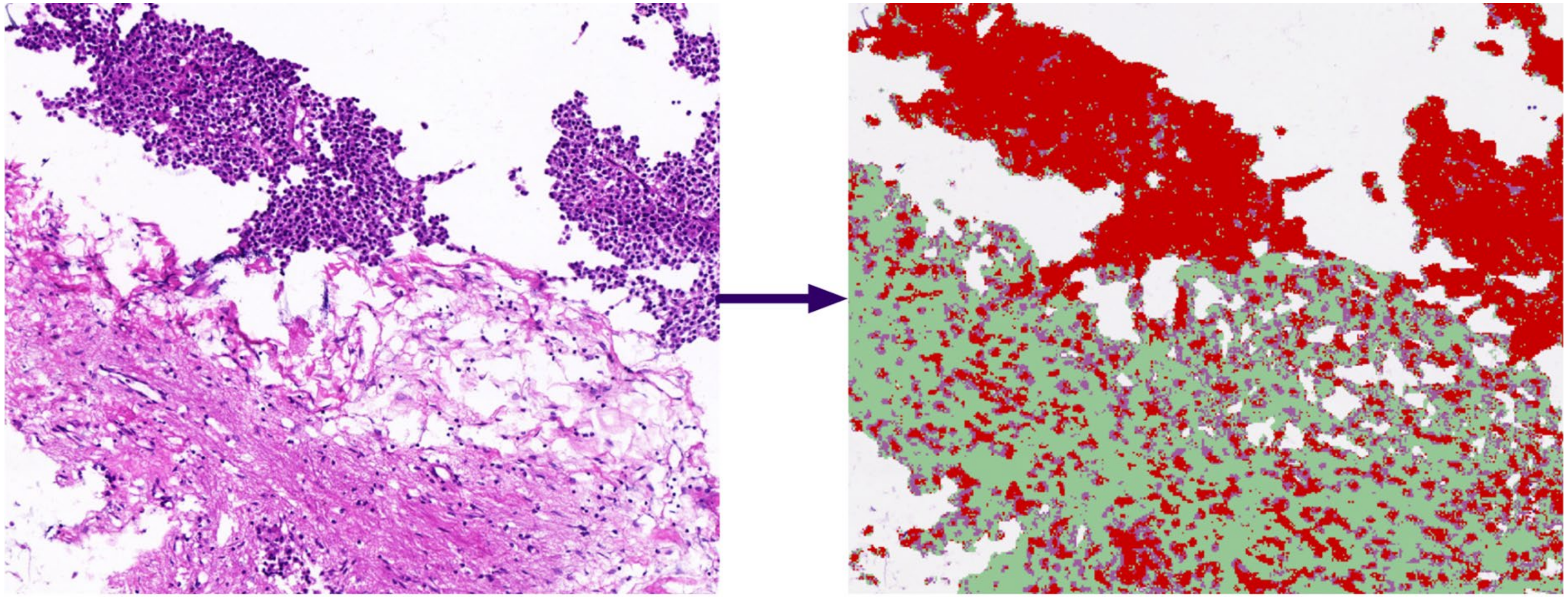

	Low-risk (N = 57)	Medium-risk/High-risk (N = 89)	
Stroma%	**14.5 ± 10.5**	**18.5 ± 12.0**	0.042
Tumor%	**58.9 ± 27.3**	**54.5 ± 22.2**	0.338

Tumor

Stroma

Figure S3. Tumors in the medium-risk and high-risk groups were associated with a higher proportion of stroma (18.5% versus 14.5%). A cell detection algorithm was used to segment the tumor area (red) and the stroma area (green) within the H&E-stained whole slide images.

Table S1. Feature comparison in morphology clusters (MC)1 and 2.

n	MC1	MC2	*p*	*p* adjusted
	N = 119	*N* = 27		
classify_blood_stroma..Immune.cells.. (mean (SD))	27.289 (19.876)	25.732 (16.254)	0.70525507	NS
classify_blood_stroma..Stroma.. (mean (SD))	17.299 (12.355)	15.307 (6.876)	0.42029631	NS
classify_blood_stroma..Tumor.. (mean (SD))	55.412 (25.353)	58.961 (19.215)	0.49546742	NS
Nucleus_Area (mean (SD))	22.859 (2.477)	23.927 (1.839)	0.03667092	NS
Nucleus_Perimeter (mean (SD))	19.118 (1.216)	20.401 (0.817)	0.00000064	0.000032
Nucleus_Circularity (mean (SD))	0.784 (0.039)	0.719 (0.020)	<0.00000001	<0.001
Nucleus_Maxcaliper (mean (SD))	7.092 (0.476)	7.715 (0.301)	<0.00000001	<0.001
Nucleus_Mincaliper (mean (SD))	4.439 (0.251)	4.408 (0.174)	0.54775378	NS
Nucleus_Eccentricity (mean (SD))	0.719 (0.034)	0.776 (0.010)	<0.00000001	<0.001
Cell_Area (mean (SD))	93.321 (14.731)	100.332 (6.931)	0.01728241	NS
Cell_Perimeter (mean (SD))	36.895 (2.752)	38.593 (1.278)	0.00216573	NS
Cell_Circularity (mean (SD))	0.821 (0.009)	0.811 (0.004)	0.00000005	0.0000025
Cell_Maxcaliper (mean (SD))	13.481 (0.967)	14.157 (0.463)	0.00054965	0.0274825
Cell_Mincaliper (mean (SD))	9.280 (0.721)	9.597 (0.316)	0.02730723	NS
Cell_Eccentricity (mean (SD))	0.679 (0.009)	0.693 (0.005)	<0.00000001	<0.001
Nucleus_Cell_ratio (mean (SD))	0.257 (0.033)	0.244 (0.013)	0.04170967	NS
Nucleus_Area_25 (mean (SD))	15.065 (1.428)	14.639 (1.025)	0.14489208	NS
Nucleus_Perimeter_25 (mean (SD))	15.070 (0.833)	15.486 (0.529)	0.01427621	NS
Nucleus_Circularity_25 (mean (SD))	0.688 (0.051)	0.610 (0.025)	<0.00000001	<0.001
Nucleus_Maxcaliper_25 (mean (SD))	5.508 (0.340)	5.821 (0.199)	0.00000943	0.0004715
Nucleus_Mincaliper_25 (mean (SD))	3.614 (0.199)	3.429 (0.118)	0.00000828	0.000414

Nucleus_Eccentricity_25 (mean (SD))	0.615 (0.046)	0.695 (0.014)	<0.00000001	<0.001
Cell_Area_25 (mean (SD))	63.746 (11.189)	67.898 (4.308)	0.06046042	NS
Cell_Perimeter_25 (mean (SD))	31.352 (2.705)	32.633 (1.047)	0.01692556	NS
Cell_Circularity_25 (mean (SD))	0.776 (0.009)	0.763 (0.005)	<0.00000001	<0.001
Cell_Maxcaliper_25 (mean (SD))	11.477 (0.977)	11.977 (0.385)	0.0101929	NS
Cell_Mincaliper_25 (mean (SD))	7.666 (0.658)	7.903 (0.253)	0.06866676	NS
Cell_Eccentricity_25 (mean (SD))	0.592 (0.012)	0.607 (0.006)	<0.00000001	<0.001
Nucleus_Cell_ratio_25 (mean (SD))	0.191 (0.031)	0.178 (0.012)	0.03158847	NS
Nucleus_Area_75 (mean (SD))	27.208 (3.477)	28.935 (2.572)	0.0162427	NS
Nucleus_Perimeter_75 (mean (SD))	21.569 (1.693)	23.508 (1.146)	0.00000008	0.000004
Nucleus_Circularity_75 (mean (SD))	0.900 (0.035)	0.841 (0.020)	<0.00000001	<0.001
Nucleus_Maxcaliper_75 (mean (SD))	8.114 (0.640)	8.971 (0.414)	<0.00000001	<0.001
Nucleus_Mincaliper_75 (mean (SD))	5.027 (0.353)	5.079 (0.245)	0.47050921	NS
Nucleus_Eccentricity_75 (mean (SD))	0.848 (0.027)	0.889 (0.008)	<0.00000001	<0.001
Cell_Area_75 (mean (SD))	114.813 (19.107)	123.914 (9.646)	0.01758062	NS
Cell_Perimeter_75 (mean (SD))	41.630 (3.048)	43.656 (1.653)	0.00107193	NS
Cell_Circularity_75 (mean (SD))	0.875 (0.010)	0.868 (0.004)	0.0011454	NS
Cell_Maxcaliper_75 (mean (SD))	15.158 (1.019)	15.952 (0.582)	0.00014529	0.0072645
Cell_Mincaliper_75 (mean (SD))	10.683 (0.880)	11.076 (0.419)	0.0252452	NS
Cell_Eccentricity_75 (mean (SD))	0.789 (0.008)	0.802 (0.004)	<0.00000001	<0.001
Nucleus_Cell_ratio_75 (mean (SD))	0.316 (0.040)	0.300 (0.017)	0.05270529	NS

Table S2. Characteristics among different immunostaining subtypes.

	GH	GH+PRL	GH+PRL+TSH	GH+TSH	Pit-1	PRL	PRL+TSH	TSH	*p*
N	24	31	20	20	23	13	7	8	
Huge	0 (0.0%)	0 (0.0%)	4 (20.0%)	2 (10.0%)	8 (34.8%)	1 (7.7%)	3 (42.9%)	1 (12.5%)	0.001
Knosp 4	0 (0.0%)	2 (6.5%)	7 (35.0%)	0 (0.0%)	4 (17.4%)	2 (15.4%)	1 (14.3%)	0 (0.0%)	0.046
Invasive	0 (0.0%)	3 (9.7%)	3 (15.0%)	3 (15.0%)	5 (21.7%)	2 (15.4%)	2 (28.6%)	1 (12.5%)	0.414
Multiple surgeries	0 (0.0%)	1 (3.2%)	5 (25.0%)	5 (25.0%)	6 (26.1%)	0 (0.0%)	2 (28.6%)	2 (25.0%)	0.015
High Ki-67	1 (4.2%)	0 (0.0%)	0 (0.0%)	1 (5.0%)	5 (21.7%)	0 (0.0%)	0 (0.0%)	0 (0.0%)	0.010
Tenacious texture	5 (20.8%)	6 (19.4%)	8 (40.0%)	9 (45.0%)	7 (30.4%)	1 (7.7%)	2 (28.6%)	3 (37.5%)	0.244
Subtotal	3 (12.5%)	5 (16.1%)	4 (20.0%)	3 (15.0%)	8 (34.8%)	1 (7.7%)	1 (14.3%)	1 (12.5%)	0.500

Table S3. Characteristics among different WHO subclassifications.

	Somatotroph	Lactotroph	Mammosomatotroph	Thyrotroph	Mature	NOS	Immature	
N	29	10	19	9	38	15	26	
Huge	1 (3.5%)	1 (10.0%)	0 (0.0%)	1 (11.1%)	2 (5.3%)	3 (20.0%)	11 (42.3%)	<0.001
Knosp 4	4 (13.8%)	2 (20.0%)	2 (10.5%)	0 (0.0%)	1 (2.6%)	3 (20.0%)	4 (15.4%)	0.481
Invasive	0 (0.0%)	2 (20.0%)	1 (5.3%)	0 (0.0%)	2 (5.3%)	4 (26.7%)	10 (38.5%)	<0.001
Multiple surgeries	5 (17.3%)	0 (0.0%)	1 (5.3%)	0 (0.0%)	4 (10.5%)	3 (20.0%)	8 (30.8%)	0.519
High Ki-67	2 (6.9%)	0 (0.0%)	1 (5.3%)	0 (0.0%)	0 (0.0%)	0 (0.0%)	4 (15.4%)	0.112
Tenacious texture	6 (20.7%)	3 (30.0%)	2 (10.5%)	2 (22.2%)	10 (26.3%)	6 (40.0%)	12 (46.2%)	0.164
Subtotal	3 (10.3%)	2 (20.0%)	5 (26.3%)	0 (0.0%)	2 (5.3%)	3 (20.0%)	11 (42.3%)	0.004
5-year recurrence-free survival	67.1% [46.0–97.8%]						53.3% [31.9–89.0%]	0.066

NOS, not otherwise specified, the pathologists do not have access to ancillary tools to enable subtyping

Table S4. Hazard ratios using other classification methods based on immunostaining.

	High-risk and medium-risk versus low-risk	
	Hazard Ratio	*p*
H score	1.97 (0.63–6.19)	0.247
Percentage	2.59 (0.84–7.96)	0.097
